# Lymphatic Filariasis Control in Tanzania: Effect of Repeated Mass Drug Administration with Ivermectin and Albendazole on Infection and Transmission

**DOI:** 10.1371/journal.pntd.0000696

**Published:** 2010-06-01

**Authors:** Paul E. Simonsen, Erling M. Pedersen, Rwehumbiza T. Rwegoshora, Mwelecele N. Malecela, Yahya A. Derua, Stephen M. Magesa

**Affiliations:** 1 DBL – Centre for Health Research and Development, Faculty of Life Sciences, University of Copenhagen, Frederiksberg, Denmark; 2 Amani Medical Research Centre, National Institute for Medical Research, Muheza, Tanzania; 3 National Institute for Medical Research, Dar es Salaam, Tanzania; Centers for Disease Control and Prevention, United States of America

## Abstract

**Background:**

In most countries of sub-Saharan Africa the control of lymphatic filariasis (LF) is based on annual mass drug administration (MDA) with a combination of ivermectin and albendazole, in order to interrupt transmission. Here we present the first detailed study on the effect of 3 repeated MDAs with this drug combination, as implemented by the Tanzanian National Lymphatic Filariasis Elimination Programme (NLFEP).

**Methodology/Principal Findings:**

Infection and transmission was monitored during a five-year period (one pre-intervention and four post-intervention years) in a highly endemic community (Kirare village) in north-eastern Tanzania. The vectors were *Anopheles gambiae*, *An. funestus* and *Cx. quinquefasciatus*. After start of intervention, human microfilaraemia initially decreased rapidly and statistically significant (prevalence by 21.2% and 40.4%, and mean intensity by 48.4% and 73.7%, compared to pre-treatment values after the first and second MDA, respectively), but thereafter the effect levelled off. The initial decrease in microfilaraemia led to significant decreases in vector infection and vector infectivity rates and thus to a considerable reduction in transmission (by 74.3% and 91.3% compared to pre-treatment level after first and second MDA, respectively). However, the decrease in infection and infectivity rates subsequently also levelled off, and low-level transmission was still noted after the third MDA. The MDAs had limited effect on circulating filarial antigens and antibody response to Bm14.

**Conclusion/Significance:**

Critical issues that may potentially explain the observed waning effect of the MDAs in the later study period include the long intervals between MDAs and a lower than optimal treatment coverage. The findings highlight the importance of ongoing surveillance for monitoring the progress of LF control programmes, and it calls for more research into the long-term effect of repeated ivermectin/albendazole MDAs (including the significance of treatment intervals and compliance), in order to optimize efforts to control LF in sub-Saharan Africa.

## Author Summary

Lymphatic filariasis (LF) is a disabling mosquito borne parasitic disease and one of the major neglected tropical diseases. In most countries of Sub-Saharan Africa the control of LF is based on yearly mass drug administration (MDA) with a combination of ivermectin and albendazole, in order to interrupt transmission. We monitored the effect of 3 repeated MDAs with this drug combination, as implemented by the Tanzanian National Lymphatic Filariasis Elimination Programme, on human infection and mosquito transmission during a five-year period (one pre-intervention and four post-intervention years) in a highly endemic community in north-eastern Tanzania. After start of intervention, human infection with the blood-stage larva of the parasite (microfilaria) initially decreased rapidly, leading to considerable reduction in transmission. The effects thereafter levelled off and transmission still occurred at low level after the third MDA. The MDAs had limited effect on molecular markers of adult worm burden (circulating filarial antigens) and transmission exposure (antibodies to Bm14 antigen) in the human population. The study highlights the importance of monitoring and regular evaluation in order to make evidence based programme adjustments, and it points to a need for further assessment of the long-term effect of repeated ivermectin/albendazole MDAs (including the importance of application intervals and treatment coverage), in order to optimize efforts to control LF in sub-Saharan Africa.

## Introduction

Lymphatic filariasis (LF), resulting from infection with the mosquito borne filarial nematode *Wuchereria bancrofti*, is a disfiguring and disabling disease. It is widespread and a major health problem in many developing countries and it is one of the most prevalent of the neglected tropical diseases. Recent estimates indicate that more than 1 billion people live in endemic areas and are at risk of infection, and more than one third of these are in Sub-Saharan Africa [1]. During the last decade, forces have been united internationally in the fight against LF through the formation of the Global Programme to Eliminate LF (GPELF) which provides guidance and support to national control programmes with an anchorage in the World Health Organization [2], [3].

The principal measure currently recommended for LF control is annual community-wide mass drug administration (MDA) of two-drug combinations to identified communities in endemic areas [2], [4]–[6]. In most endemic countries a combination of diethylcarbamazine (DEC) and albendazole is used, but due to the risk of serious adverse reactions in individuals infected with *Onchocerca volvulus*, a combination of ivermectin and albendazole is used in African countries which are co-endemic for onchocerciasis. Although the MDAs rarely completely clear the *W. bancrofti* infection from the treated individual, it is assumed that the reduction in the microfilarial load in the endemic population will lead to a simultaneous reduction – or even elimination – of transmission. Hence the term “transmission interruption” has been adopted for this strategy. Monitoring and regular evaluation of the effect of the control efforts is important in order to be able to quantify the impact, to make evidence based programme adjustments, and to eventually make a decision to stop the activities when the goal has been reached [7], [8].

LF is widespread in Tanzania. Particular high endemicity is seen along the coast of the Indian Ocean and in areas adjacent to the great lakes. Tanzania was one of the first countries in Africa to initiate implementation of control, with the Tanzanian National Lymphatic Filariasis Elimination Programme (NLFEP) being launched in 2000 [9]. The goal of the NLFEP is to apply annual MDA with a combination of ivermectin (150–200 µg/kg) and albendazole (400 mg) to all individuals aged 5 years and above in selected programme areas. It has previously been shown in Tanzania that this treatment regimen drastically reduce the *W. bancrofti* microfilarial load [10].

Tanga Region, located in the north-eastern part of Tanzania, was enrolled in the NLFEP and received the first MDA in October 2004. A study to monitor the effect of the programme in a highly endemic community in this region (Kirare village) was initiated in October 2003 (one year before the planned start of MDAs) in order to obtain baseline data on infection and transmission before start of control activities and to subsequently monitor the effect of control. Here we report on the effect of the first three rounds of MDA given by the Tanzanian NLFEP on infection and transmission of LF in Kirare village. To our knowledge this is the first detailed assessment of the effect of an ivermectin/albendazole based MDA programme for LF control in Sub-Saharan Africa.

## Methods

### Study design

The main part of the study was conducted in Kirare, a village located approximately 20 km south of Tanga town (Tanzania) along the Tanga-Pangani road, in an area known to be highly endemic for LF [10], [11]. Prior to the study there had been no attempts to control LF in the study site, and antifilarial drugs had not been available.

The LF endemic districts of Tanga Region were included in the NLFEP and received the first round of MDA in October 2004. It was the intention that MDAs should be implemented during every month of October in subsequent years, but logistic and financial constraints lead to delays. The next two MDAs took place in February 2006 and May 2007, while the fourth MDA was scheduled for November 2008. The present paper reports on the effect of the first three MDAs, until October 2008.

In order to get a year of baseline information about vectors and transmission before the first MDA, weekly entomological surveillance covering 50 households was initiated in Kirare in November 2003. This activity has continued uninterrupted since then. A cross-sectional examination for clinical manifestations related to LF and for *W. bancrofti* microfilariae (mf) covering individuals ≥1 year was carried out during the month prior to the first MDA. It was attempted to make similar cross-sectional surveys for mf in the month prior to subsequent MDAs, but due to postponements of the MDAs, the next mf examinations of the human population took place in January 2006, January 2007, October 2007 and October 2008. During the cross-sectional surveys, individuals living in the households used for mosquito collections were requested to provide a venous blood sample for serology.

### Ethics

Village meetings were held before and regularly during the study period in the national language (Swahili, which is widely spoken and understood in the area) to inform the villagers about the study contents and implications and to obtain their cooperation. The meetings included information about individuals' right to withdraw from participation during any part of the study without negative consequences. Prior to any blood sampling or clinical examination, the individual was asked if the purpose and consequences as explained during the meetings had been understood, and questions for further clarification were answered. Their oral consent to participate (from adults, and from parents or guardians of individuals less than 15 years old) was recorded on the survey form. Oral consent is the traditional way for making agreements in the study area, whereas written consent is unfamiliar and would cause suspicion and refusal to participate. Permission and ethical clearance to carry out the study (including the use of oral informed consent) was granted by the Medical Research Coordinating Committee of the National Institute for Medical Research (NIMR) in Tanzania and the study protocol was reviewed by the Danish National Committee on Biomedical Research Ethics.

### Entomological surveillance

The village houses were mapped and the name, age and sex of villagers were registered. The households were divided into 50 groups of nearly equal size and one household from each group was randomly selected for surveillance of vectors and transmission. If the selected household had only one inhabitant it was de-selected, and the nearest household to it that held at least two inhabitants was selected instead. From the beginning of November 2003, mosquitoes were collected from each of the selected households once weekly (10 houses were sampled on the night of each weekday) as described previously [12]. Briefly, on the night of collection, a battery-operated CDC light trap was placed beside an occupied bed provided with an un-impregnated mosquito net. The traps were turned on at 18.00 hours and off at 06.00 hours. Mosquitoes were collected from the traps in the morning and brought to the laboratory in Tanga. They were knocked down with chloroform, sorted and identified to species by morphology. Live female mosquitoes were dissected and examined for the first-stage, second-stage and human infective third-stage larvae of *W. bancrofti*. To cross-check the results of the initial infection examinations, all larvae-positive slides and 20% of the negative slides were stained in Mayer's acid haemalum and later re-examined.

### Examination of the human population

Examinations were carried out during the evening. Clinical examination by an experienced clinician started at 20.00 hours. Signs of chronic LF were graded as described [13], but in this presentation lymphedema/elephantiasis ≥ grade I (loss of contour, pitting edema) and hydrocele ≥ grade II (true hydrocele ≥6 cm, with fluid accumulation) are reported as elephantiasis and hydrocele, respectively. Blood sampling for quantification of mf started at 21.00 hours. From each individual, 100 µl of finger prick blood was collected in a heparinised capillary tube and transferred to a tube with 900 µl of 3% acetic acid. Later in the laboratory, the specimens were transferred to a counting chamber and examined for mf under a compound microscope [14]. All specimens were blindly examined twice by two different technicians, and the mean count was recorded as the mf intensity.

Additional venous blood samples of 5 ml were collected from volunteers from the 50 mosquito collection houses immediately after the finger prick blood sampling. Specimens were collected in plain vacutainer tubes, which were left overnight at 4°C for the blood to clot. Serum was collected the following day and stored at −20°C until use.

### Assessment of treatment coverage and bed net use

The treatment coverage was assessed in two different ways. First, the official programme coverage for Kirare Ward (which in addition to Kirare also includes another slightly larger neighbouring village) was obtained from the regional NLFEP office in Tanga (‘reported coverage’). This had been calculated as the recorded number of treatments given in the ward by the programme divided by the registered population ≥5 years for the ward. Second, the study conducted a questionnaire survey in the village within one month after each MDA, during which individuals ≥5 years were asked whether or not they had taken the treatment (‘surveyed coverage’).

At the time of the start of the study in 2003, very few houses in Kirare possessed a bed net. However, it was observed that bed nets gradually became more common, and a questionnaire survey carried out in June 2007 (together with the survey for MDA coverage) indicated that the number of bed nets available in the households of Kirare relative to the number of study individuals amounted to 18.6%.

### Tests for circulating filarial antigens and antibodies to Bm14

Serum samples were examined in the laboratory in Denmark. Before handling and testing of sera, 3 µl/ml TNBP (tri-n-butyl-phosphate, Sigma T-4908) and 10 µl/ml Tween 80 (polyoxyethylenesorbitan, Sigma P-1754) were added to the specimens to inactivate lipid coated vira [15].

The specimens were examined for specific circulating filarial antigen (CFA) by using the TropBio enzyme linked immunosorbent assay (ELISA) kit for serum specimens (catalog no. 03-010-01; TropBio Ltd., Pty., Townsville, Australia). The test was performed according to procedures from the manufacturer. Serum specimens with a response ≥ that of the enclosed standard 2 (32 CFA units) were considered positive for CFA.

The specimens were moreover examined by ELISA for IgG4 antibodies to the recombinant antigen Bm14, supplied by the NIH/NIAID Filariasis Research Repository Center (FR3; Smith College, Massachusetts, USA). The ELISA was performed according to procedure enclosed with the supplied antigen. All incubations were carried out at room temperature on a shaker. Briefly, ELISA plates (Maxisorp F96 immunoplates, Nunc, Denmark) were coated overnight with 100 µl diluted antigen (2 µg Bm14 per ml in carbonate buffer, pH 9.6). The plates were then washed 3 times with PBS/0.5% Tween 20, blocked by adding 200 µl/well of the same buffer containing 5% heat inactivated foetal calf serum (FCS) for 30 minutes, and washed 3 times. Plates were thereafter incubated for 1 hour with 100 µl of the human test serum diluted 1∶500 with PBS/Tween/FCS, washed 3 times with PBS/Tween, incubated for 1 hour with 100 µl horse-radish peroxidase-labelled mouse-anti-human IgG4 (M1331, Sanquin, Amsterdam, Netherlands) diluted 1∶2000 with PBS/Tween/FCS, and washed 3 times with PBS/Tween. Finally, 100 µl OPD substrate solution, prepared from OPD tablets as instructed by the manufacturer (Dako, Denmark) was added to each well, the plate was incubated for 10 minutes in the dark, and the optic density (OD value) was read at 490 nm on an ELISA reader (Microplate reader 550, Bio-Rad, USA). Serum specimens with an OD-value higher than the mean plus four standard deviations of OD-values for five control individuals from a non-endemic area (Denmark) were considered positive for IgG4 to Bm14.

In all ELISAs (for CFA and IgG4) specimens were tested in duplicate (arithmetic mean of the two tests was used as the result), and all sera from the same person were tested on the same ELISA plate.

### Data analysis

The mf intensities were adjusted for sampling time by multiplying the counts with a time-specific factor. A list of the adjustment factors for every 10 minutes between 20.00 and 02.00 hours, and the method for deriving these on the basis of a detailed analysis of the mf periodicity curve, were presented previously [16]. Geometric mean intensities (GMIs) of microfilaraemia, CFA intensity and Bm14 antibody level were calculated as antilog ((∑ log x+1)/n)−1, with x being the mf/ml, CFA units or OD-values, respectively, and n the number of individuals included.

Only individuals registered as inhabitants in Kirare during the pre-MDA survey were included in the post-MDA follow-up surveys (i.e. newborn or immigrants were not included).

The entomological indices for vector biting and transmission were calculated as previously [12]. Briefly, the monthly biting rate (MBR; a measure of the number of mosquito bites per person in the month) was calculated as: (total mosquito catch × days in month ×3)/(number of catching nights × number of light traps ×2). The monthly transmission potential (MTP; a measure of the number of infective larvae to which a person is exposed in the month) was calculated as: (MBR × total number of infective larvae seen in the dissections)/(number of mosquitoes dissected). The ‘infectivity rate’ was calculated as the percent of mosquitoes infected with infective larvae (L3) and the ‘infection rate’ as the percent of mosquitoes infected with any stage of the parasite (L1, L2 and/or L3).

## Results

### Lymphatic filariasis in the community before treatment

During the baseline surveys in September 2004, a total of 1,112 individuals aged 1 year and above were registered in Kirare (Table 1). Among these, the male to female ratio was 0.96 and 51.7% were less than 20 years old.

**Table 1:** *Wuchereria bancrofti* microfilaraemia in Kirare village as seen during the pre-MDA survey in September 2004.

Age group in years	No. registered	No. examined for mf (%*)	No. positive for mf (%**)	Mf GMI among all examined#	Mf GMI among positive#
1–4	134	116 (86.6)	1 (0.9)	0.04	70.0
5–9	190	165 (86.8)	22 (13.3)	1.30	721.2
10–14	177	154 (87.0)	43 (27.9)	5.68	896.0
15–19	74	52 (70.3)	15 (28.8)	5.78	760.9
20–29	148	111 (75.0)	42 (37.8)	10.49	634.0
30–39	137	115 (83.9)	37 (32.2)	7.12	670.3
40–49	89	74 (83.1)	21 (28.4)	5.78	849.6
50+	163	132 (81.0)	44 (33.3)	9.60	1190.0
Total	1112	919 (82.6)	225 (24.5)	4.11	781.3

*) of registered.

**) of examined.

# ) In mf/ml blood.

A total of 919 (82.6%) individuals had their blood examined for mf, and 225 (24.5%) were mf positive (Table 1). The prevalence increased rapidly by age in the younger age groups, until a level was reached during late teenage years. It was significantly higher among males than among females (27.7% vs. 21.7%; chi-square test, P<0.05), and this trend was seen for all age groups. The mf intensities among mf-positive individuals ranged from 10 to 25,370 mf/ml blood. The overall mf GMIs were 4.11 mf/ml when calculated for all examined individuals and 781.3 mf/ml when calculated for mf positive individuals only (Table 1). These indices were also significantly higher for males than for females (6.10 vs. 2.82 and 1192.4 vs. 486.1; t-test, P = 0.002 and P<0.001, respectively). Chronic manifestations of filariasis were common in Kirare. Thus, 4.1% of the examined adults (≥20 years old) had elephantiasis of the leg, and 32.8% of adult males had hydrocele.

**Figure 1:**
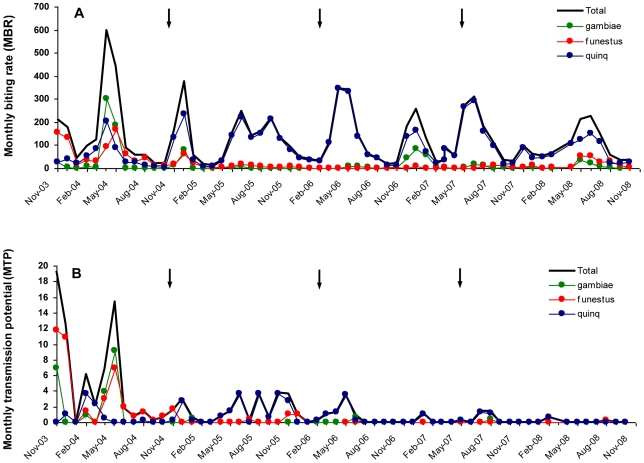
Seasonal variation in monthly biting rate (MBR) and monthly transmission potential (MTP) in Kirare village during the study period (November 2003 to October 2008). MBR, panel A; MTP, panel B. Thick black line  =  all vector species combined; Thin green line  =  *Anopheles gambiae*; Thin red line  =  *An. funestus*; Thin blue line  =  *Culex quinquefasciatus*. Arrows indicate rounds of mass drug administration.

Dissection of the mosquitoes caught from the 50 collection houses during the 11 months pre-MDA period indicated that *An. funestus*, *An. gambiae* and *Cx. quinquefasciatus* were vectors of *W. bancrofti* (Table 2). Among these, *An. funestus* was the most abundant (mean MBR of 71.3), had the highest *W. bancrofti* infection prevalence (infectivity rate of 2.3%; infection rate of 4.5%) and contributed most to transmission (mean MTP of 3.5), followed by *An. gambiae* (49.3, 1.4%, 3.8% and 1.9, respectively) and *Cx. quinquefasciatus* (53.6, 0.5%, 2.0% and 0.7, respectively). Overall, the two Anopheline vectors were responsible for 88.5% of the transmission during the pre-MDA period. Vector biting and parasite transmission varied by season (Figure 1), but was generally most intense during and shortly after the rainy seasons (months of November/December and March/April/May).

**Table 2:** Vector mosquito catches from the 50 collection houses in Kirare village, and the results of dissections, during the pre-MDA period and the three post-MDA periods.

	Total	*An. gambiae*	*An. funestus*	*Cx. quinquefasciatus*
Pre-MDA period (11 months)*				
No. mosquitoes caught	8346	2335	3385	2626
No. mosquitoes dissected	5396	1477	2080	1839
No. with infection (% of dissected)	187 (3.5)	56 (3.8)	94 (4.5)	37 (2.0)
No. with L3 (% of dissected)	77 (1.4)	20 (1.4)	48 (2.3)	9 (0.5)
No. of L3	153	51	87	15
Post-MDA period 1 (16 months)**				
No. mosquitoes caught	8872	527	929	7416
No. mosquitoes dissected	7840	338	705	6797
No. with infection (% of dissected)	128 (1.6)	7 (2.1)	20 (2.8)	101 (1.5)
No. with L3 (% of dissected)	35 (0.4)	1 (0.3)	6 (0.9)	28 (0.4)
No. of L3	101	1	15	85
Post-MDA period 2 (15 months)***				
No. mosquitoes caught	7849	970	75	6804
No. mosquitoes dissected	7071	790	67	6214
No. with infection (% of dissected)	22 (0.3)	3 (0.4)	0 (0.0)	19 (0.3)
No. with L3 (% of dissected)	12 (0.2)	1 (0.1)	0 (0.0)	11 (0.2)
No. of L3	34	3	0	31
Post-MDA period 3 (18 months)****				
No. mosquitoes caught	9892	631	1148	8113
No. mosquitoes dissected	9088	564	848	7676
No. with infection (% of dissected)	72 (0.8)	7 (1.2)	4 (0.5)	61 (0.8)
No. with L3 (% of dissected)	10 (0.1)	1 (0.2)	1 (0.1)	8 (0.1)
No. of L3	17	1	1	15

*Nov-03 to Sep-04;

**Oct-04 to Jan-06;

***Feb-06 to Apr-07;

****May-07 to Oct-08.

### MDA treatment coverage

Treatment coverage during the three MDAs, as assessed separately by the NLFEP (‘reported coverage’) and the study (‘surveyed coverage’), are shown in Table 3. The results from the two assessment methods differed considerably. This was most marked for the first MDA in October 2004 during which surveyed coverage was higher (82.3%) than reported coverage (62.1%). For the next two MDAs in February 2006 and May 2007, reported coverages were higher than surveyed coverages (94.8% vs. 79.0% and 80.0% vs. 69.9%, respectively).

**Table 3:** Treatment coverage during mass drug administration (MDA) 1, 2 and 3.

MDA no.	Time of MDA	Treatment coverage in %	
		Reported coverage*	Surveyed coverage**
1	Oct-04	62.1	82.3
2	Feb-06	94.8	79.0
3	May-07	80.0	69.9

*) Official NLFEP coverage for eligible individuals in Kirare Ward (include Kirare village plus one neighboring village of slightly larger size).

**) Interview of individuals ≥5 years (at time of MDA) carried out within one month after treatment. 99.7%, 100.0% and 81.0% of these were interviewed during MDA 1, 2 and 3, respectively.

### Effect of MDAs on microfilaraemia

**Figure 2:**
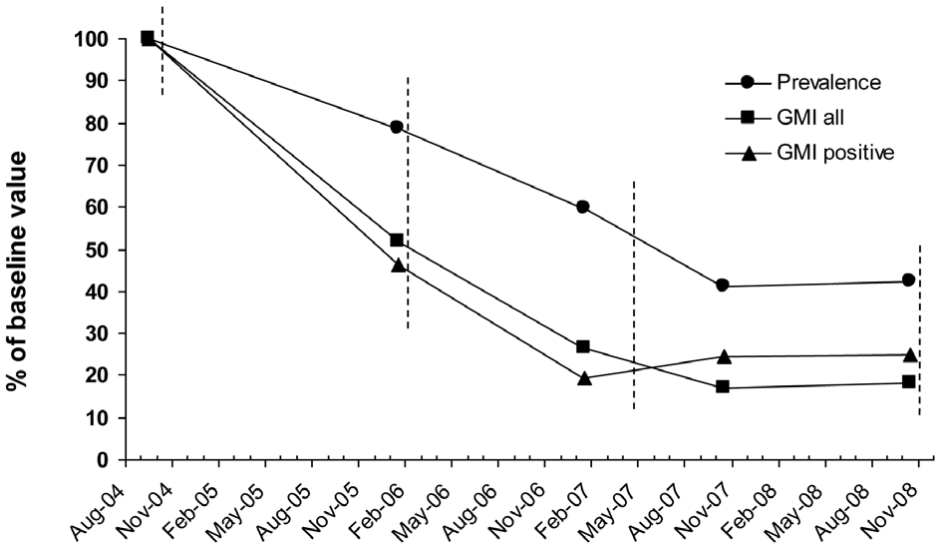
The effect of three rounds of mass drug administration (MDA) on *Wuchereria bancrofti* microfilaemia in Kirare village. Lines indicate microfilaria (mf) prevalence (•), mf geometric mean intensity (GMI) among all examined individuals (▪) and mf GMI among positive individuals only (▴). All indices are expressed in % of the baseline value. Vertical stippled lines indicate rounds of MDA.

The effect of the MDAs on microfilaraemia is shown in Table 4 and Figure 2. Although the survey coverage decreased from one survey to the next, the mean age remained stable (range of means (± standard deviation): 24.3±20.2 to 25.4±20.8). Following each MDA the mf prevalence decreased progressively, from 24.5% in the pre-MDA survey (September 2004) to 10.1% five months after the third MDA (October 2007). Statistical analysis of data from individuals examined at both time points indicated significant decreases in mf prevalence from surveys 1 to 2, 2 to 3 and 3 to 4, but not from survey 3 to 5 (n = 709, 655, 606 and 491; McNemar's chi-square test for paired proportions, P<0.001, P<0.001, P = 0.002 and P = 0.14, respectively). During the one-year period without treatment, from October 2007 to October 2008, there was a trend towards an increase in prevalence from 10.1 to 10.6%, but the difference was not statistically significant (n = 458; P = 0.20).

**Table 4:** Microfilaraemia in Kirare village during the pre-MDA survey (September 2004) and the four post-MDA surveys (January 2006, January 2007, October 2007 and October 2008).

Survey no.	Time of survey	No. examined (%)	No. positive for mf (%)	Mf GMI for all examined*	Mf GMI for positives*
1	Sep-04	919 (82.6)	225 (24.5)	4.11	781.3
2	Jan-06	798 (71.8)	154 (19.3)	2.12	360.4
3	Jan-07	831 (74.7)	121 (14.6)	1.08	150.7
4	Oct-07	674 (60.6)	68 (10.1)	0.70	190.8
5	Oct-08	565 (50.8)	60 (10.6)	0.75	194.7

The target population for all surveys was the 1112 individuals aged ≥1 year in at the pre-MDA survey.

*) In mf/ml blood.

A similar trend was seen for the mf GMIs calculated on the basis of all examined individuals, but the reductions observed during the first three periods were proportionately greater than the reductions seen for the prevalences (reached 17.0% of pre-MDA values in October 2007). Statistical analysis of data from individuals examined at both time points again indicated significant decreases from surveys 1 to 2, 2 to 3 and 3 to 4, but not from survey 3 to 5 (paired t-test, P<0.001, P<0.001, P = 0.007 and P = 0.30, respectively). Also, a small but statistically insignificant increase was seen in this parameter between October 2007 and October 2008 (paired t-test, P = 0.11).

The mf GMI among the mf positives decreased even more steeply during the first two periods (reaching 19.3% of pre-MDA values in January 2007). It thereafter increased to 24.4% and 29.9% of pre-MDA values in October 2007 and October 2008, respectively. However, in analyses of data from individuals who were positive during both examinations the increases did not reach statistical significance, neither between January and October 2007, nor between January 2007 and October 2008 (n = 43 and 40; Paired t-test, P = 0.40 and 0.19, respectively).

### Effect of MDAs on vectors and transmission

**Figure 3:**
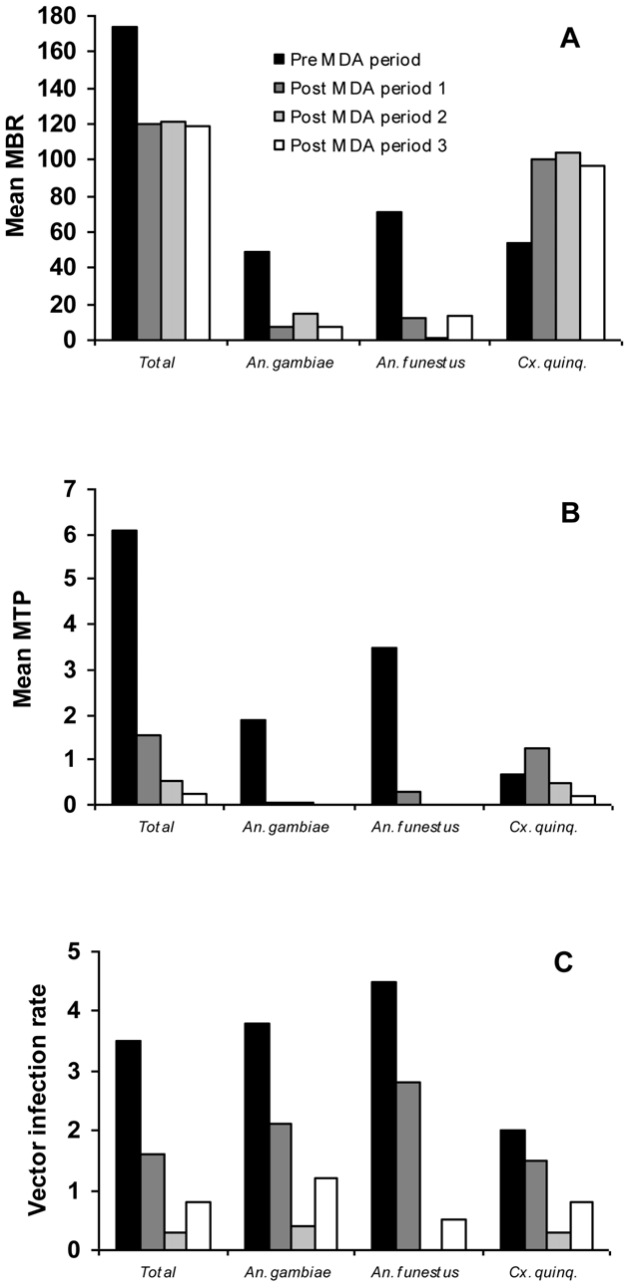
Mean monthly biting rate. (MBR; A), mean monthly transmission potential (MTP; B) and vector infection rate (C) in Kirare village during the pre-MDA period and the three post-MDA periods.

The vector species composition and vector density changed considerably from the pre-MDA to the three post-MDA periods (Table 2, Figure 3A). Anopheline vectors predominated during the pre-MDA period, with *An. gambiae* and *An. funestus* comprising 68.5% of the catch, while *Cx. quinquefasciatus* predominated and comprised 84.1% of the catch during the three post-MDA periods combined (chi-square test, P<0.001). The overall mean MBR was reduced from 174.3 during the pre-MDA period to 119.8 during the three post-MDA periods. As discussed later, these changes between pre- and post-MDA periods were probably not related to the MDAs.

For the vector species combined, the start of MDA led to a significant decrease in the vector infectivity rate, from 1.43% of vectors containing infective larvae in the pre-MDA period to 0.45% and 0.17% in the first and second post-MDA periods, respectively (chi-square test, P<0.001 and P = 0.004, respectively). In the third post-MDA period, this trend continued (to 0.11%) but the decrease was statistically insignificant (chi-square test, P = 0.42). Similar trends of decrease in infectivity rate were seen for the individual vector species (Table 2). These decreases resulted in a considerable and progressive reduction in transmission of *W. bancrofti* infective larvae. Thus, the overall mean MTP reduced from 6.08 during the pre-MDA period to 1.56, 0.53 and 0.22 (25.7%, 8.7% and 3.6% of pre-MDA values) during the first, second and third post-MDA periods, respectively (Figure 3B). Transmission by the two Anopheline vectors was reduced to an even greater extent, and was almost undetectable during post-MDA periods 2 and 3. For *Cx. quinquefasciatus*, however, the rise in MBR after start of treatment led to an initial rise in transmission during the first post-MDA period, which was then followed by a reduction in subsequent periods. Overall, *Cx. quinquefasciatus* was responsible for most transmission after start of treatment (contributed 82.1% of the MTP during the three post-MDA periods combined).

For the vector species combined, the first and second rounds of MDA both led to significant decreases in vector infection rates (i.e. percent of vectors infected with any stage of the parasite), from 3.5% in the pre-MDA period to 1.6% and 0.31% in the first and second post-MDA periods (chi-square test, P<0.001 for both tests). However, this was followed by a significant increase to 0.79% in the third post-MDA period (chi-square test, P<0.001). Similar patterns were seen for the individual vector species (Figure 3C).

### Effect of MDAs on CFA and antibodies to Bm14

Ninety individuals from the mosquito collection houses volunteered to provide a serum specimen at the pre-MDA survey, and these were requested to also provide a specimen at the four post-MDA surveys. All specimens were analysed for CFA and IgG4 antibodies to Bm14 (Table 5). There were only limited changes in prevalence of CFA and Bm14 antibody positivity between the pre-MDA and the fourth post-MDA survey. When data from individuals examined at both time points were analysed statistically, a significant difference between pre-MDA and subsequent post-MDA examinations was found only for the decrease in CFA prevalence in October 2008 (n = 49; McNemar's test, P = 0.03).

**Table 5:** Circulating filarial antigen (CFA) and antibody to Bm14 in 90 volunteers from the mosquito collection houses in Kirare village.

Survey no.	Time of survey	No. examined	CFA			Antibody to Bm14		
			No. positive (%)	GMI for all examined*	GMI for positives*	No. Positive (%)	GMI for all examined**	GMI for positives**
1	Sep-04	90	48 (53.3)	106.9	4835	71 (78.9)	0.784	1.083
2	Jan-06	66	35 (53.0)	96.1	3106	53 (80.3)	0.642	0.854
3	Jan-07	72	37 (51.4)	79.2	2871	56 (77.8)	0.452	0.614
4	Oct-07	61	32 (52.5)	81.9	2386	48 (78.7)	0.467	0.627
5	Oct-08	49	22 (44.9)	47.3	2369	37 (75.5)	0.405	0.568

The individuals provided a serum specimen at the pre-MDA survey (September 2004)^#^ and were followed up at the four post-MDA surveys (January 2006, January 2007, October 2007 and October 2008).

# ) 55 females and 35 males; Age in 2004: 1–66 years (mean 24.1 years).

*) In CFA units.

**) In OD-values.

More pronounced decreases were seen in the mean levels of these indices with increasing numbers of MDAs. Thus, the CFA GMI among all examined individuals decreased to 44.2% of pre-MDA level (from 106.9 to 47.3 CFA units) and the Bm14 antibody OD-value among all examined decreased to 51.5% of pre-MDA level (from to 0.784 to 0.405) between the pre-MDA survey and the fourth post-MDA survey. When data from individuals examined at both time points were analysed statistically, all four post-MDA CFA GMIs were significantly lower than the pre-MDA value (n = 66, 72, 61 and 49; Paired t-test, P = 0.03, <0.001, <0.001 and <0.001, respectively), and all four post-MDA Bm14 OD-values were significantly lower than the pre-MDA value (paired t-test, P<0.001 for all tests).

## Discussion

Annual MDA with a combination of ivermectin and albendazole is recommended for the control of LF in African countries which are co-endemic for onchocerciasis [2], [4], [6]. Early trials in selected mf positive individuals [17]–[19] as well as community based field trials [10], [20]–[22] demonstrated that this drug combination safely and effectively reduced *W. bancrofti* microfilaraemias, thus making it suitable for use in large scale transmission control [23]. Here we report on the effect of repeated MDAs with this drug combination in a highly LF endemic community of Tanzania, as applied by the NLFEP.

The study village was immensely affected by LF before intervention started, in terms of infection, disease and transmission. Thus, the overall prevalence among those examined was 24.5% for mf, 53.3% for CFA, and 78.9% for antibodies to Bm14. Four percent of the adults moreover suffered from limb elephantiasis, while 32.8% of adult males had hydrocele. The vectors were identified as *An. gambiae*, *An. funestus* and *Cx. quinquefasciatus,* and the two *Anopheles* species were responsible for most of the transmission during the baseline year. The general pattern of LF in the study community thus resembled that previously reported from the same area [11]–[13], [24]. However, in contrast to experience from the earlier studies, the survey team faced reluctance from some of the villagers to participate in the cross-sectional examinations. This behaviour appeared to be related to ‘disturbances’ created by the location of the village along the main road between Tanga and Pangani and to a strong engagement of some of the villagers in local politics. Despite great efforts made, the survey coverage for blood sampling decreased from one survey to the next.

The weekly collection of vectors from the 50 selected households posed less of a challenge, as the inhabitants were generally pleased to see mosquitoes being caught and removed from their houses. The collection and dissection of vectors is a time-consuming and expensive task, which is not recommended as a routine for monitoring LF elimination programmes. However, it provides the most exact information about vector dynamics and transmission of LF, and as such is an important research tool in projects aiming to investigate the effect of control programmes in detail. A newly developed PCR technique, capable of distinguishing the different parasite stages in the vectors, may replace the laborious dissection activity in future assessments of LF transmission [25].

According to the original plan from 2004, the NLFEP should implement a MDA each year in October. However, due to the complicated logistics and limited funds available the programme (which includes a large part of the country) faced delays in activities. As a result, the three inter-MDA periods covered by the present study widened from 12 to 16, 15 and 18 months, respectively. Due to uncertainty of the timing of the MDAs, the time-interval between the cross-sectional surveys in Kirare and the MDAs moreover often became considerably longer than desired.

The treatment coverage is a key factor to consider when assessing the impact of MDAs [7], [26], [27]. However, exact information on treatment coverage is generally not easily available from large scale control programmes, and the reliability of the data may be highly variable, as personal and political interests and difficult working conditions can have a major impact on their quality. Two different approaches were used to gather information about treatment coverage in the present study. First, the official ‘reported coverage’ was obtained from the NLFEP office in Tanga Region. This is calculated on the basis of the drug distributors' recordings during drug delivery, and may to some extent be affected by the time pressure during drug distribution and the attention and honesty of the distributors. The ‘reported coverage’ moreover depends on the quality of the population data used as the denominator in the calculations. Second, a ‘surveyed coverage’ was obtained by the study by interviewing villagers individually after the MDA. The quality of this figure may be affected by memory and honesty of those interviewed. Some villagers may feel ashamed admitting that they did not take the drugs, when health messages have encouraged them to do so. This latter issue may have had the highest impact during the first MDA, when the overall concept of MDAs was new and information dissemination most intensive, and may perhaps have accounted for the large difference in treatment coverage obtained with the two approaches in 2004. Both approaches indicated a decrease in treatment coverage from the second to the third MDA, thus stressing the importance of continued dissemination of health messages encouraging individuals to participate in the MDAs [28].

The first two MDAs had a considerable and statistically significant suppressing effect on microfilaraemia in the population. Thus, at the January 2007 survey (28 months after first and 12 months after second MDA), the prevalence was reduced by 40.4%, and mf GMI for all examined was reduced by 73.7%, when compared to pre-MDA values. Following the third MDA, a further but slightly less significant reduction was seen in these parameters in October 2007 (reductions by 58.8% and 83.0%, respectively, compared to pre-MDA). During the last survey in October 2008 there was no further decrease compared to the previous survey, but rather a small but statistically insignificant increase. It may be argued that data from October 2007 were collected only 5 months after the third MDA, and that microfilaraemias could therefore be at particularly low levels due to the short time after treatment. However, even the apparent decrease in mf prevalence and mf GMI among all examined between January 2007 and October 2008 was not statistically significant. The mf GMI among mf positives showed a trend towards a increase between January 2007 and October 2008 (although still not statistically significant), suggesting that the apparent waning effect of the MDAs was most marked among individuals who had not yet cleared their mf (these individuals also had a considerable higher mean mf burden at pre-MDA than the other mf positive individuals; results not shown). Possible reasons for the levelling out of the effect of MDAs on microfilaraemia in the latter part of the study will be discussed later.

There was a clear shift in vector species composition between the pre-MDA period and the period following start of MDA, from predominantly *Anopheles* transmission to transmission by *Cx. quinquefasciatus*. An earlier study (1998–2001) carried out in a nearby village (Masaika) had also incriminated *Anopheles sp.* as the predominant vectors in the area [12], [29]. It is unlikely that the shift was related to the MDAs. The more frequent use of bed nets by the village habitants may have contributed to the shift, but bed net coverage was still rather low in 2008 and cannot have been a major reason. Rather it could be due to environmental and/or climatic changes. Examination of the monthly rainfall pattern for the area (results not shown) did not immediately reveal any obvious change during the study period, and a more detailed analysis of environmental factors (including rainfall intensity and frequency pattern, temperature variations and vegetation cover) that could be the cause of the observed change in vector populations should be carried out. It is interesting to note that a significant reduction in prevalence of malaria cases (malaria and LF share the same *Anopheles* vectors) has also been observed in coastal East Africa during the same period [30]. The observed shift in LF vector species composition also points to the importance of considering the complexity of transmission in areas with several vectors. When conditions for one vector species get worse, other vector species may thrive and take over the major part of transmission.

The MDA-induced reduction in human microfilaraemia led to a considerable decrease in transmission, which was obvious for all three vector species. However, whereas *Anopheles* transmission was almost interrupted after the second MDA, that by *Cx. quinquefasciatus* continued at low level even after the third MDA, being supported by the persisting low level of human microfilaraemia and the large number of vectors. Transmission intensity varied with the season, and during the latter period of the study almost exclusively took place during and shortly after the rainy seasons when vectors were most abundant.

The impact of the MDAs on CFA (an indirect measure of adult worm burden; [31]) and antibodies to Bm14 (an indirect measure of infection and/or exposure to transmission; [31]) was limited, and not nearly as marked as seen in programmes using the combination of diethylcarbamazine (DEC) and albendazole for control of LF [31], [32]. Thus, although the mean intensity of both indices had decreased to about half of the pre-MDA level at the end of the study in October 2008, there was no clear effect on the prevalences. Previous studies have similarly indicated a limited effect of the ivermectin/albendazole combination on circulating filarial antigenemia [10], [21], in agreement with the general understanding that ivermectin is mainly a microfilaricidal drug. The limited reduction in level of antibodies to Bm14 concurs with the observed remaining infection burden and continued transmission of LF in the study community.

The effect of the ivermectin/albendazole MDAs in this study obviously did not match the reductions in infection and transmission seen following MDAs with the DEC/albendazole combination [31]–[33]. Although the MDAs caused reduction in microfilaraemia and transmission in relation to pre-MDA levels, especially after the first two rounds, the waning effect of the MDAs observed later is a course for concern. There may be a number of reasons, which needs to be further investigated. First, the intervals between treatments were longer than the recommended one year period. An increase in both mf and CFA prevalence was recently reported after a single MDA with DEC/albendazole had been missed in Haiti [34]. As ivermectin does not kill the adult worms, new mf may start to appear soon after drug action cease. Previous studies showed that the ivermectin/albendazole combination had maximum effect on the microfilaraemia at 3–6 months after treatment [10], [20]. MDAs might therefore have to be implemented even more frequently than once yearly with this drug combination to achieve a continued long-term reduction in mf burden. Second, the treatment coverage obtained (whether ‘reported’ or ‘surveyed’) may, as argued earlier, not be a reliable measure of the proportion of individuals actually swallowing the drugs, and the ‘true’ coverage could be lower than indicated. There is a great need both for development of improved ways of assessing the ’true’ treatment coverage in MDAs and for development of better means to increase this coverage [28], [35]. Third, knowledge on the long-term effect of repeated treatment with the ivermectin/albendazole combination is limited. A study in Ghana showed a similar effect during first and second treatment (given to the same individuals with a one year interval), but it cannot be excluded that the effect may wane with the increasing number of MDAs [22].

The present study emphasizes the importance of ongoing surveillance for monitoring the progress of LF control programmes, in order to optimize control efforts in Sub-Saharan Africa. It calls for more research into the long-term effect of repeated ivermectin/albendazole MDAs (including the significance of treatment intervals and compliance), and it suggests that evidence based programme adjustments, perhaps combined with introduction of additional measures such as vector control [6], [36] will be necessary in order to reach the goal of LF elimination within a reasonable time-frame.
